# Leptomeningeal and Ependymal Seeding of a High-Grade Glioma Following Ventricular Opening and Carmustine Wafer Implantation: A Case Report Highlighting a Diagnostic Dilemma

**DOI:** 10.7759/cureus.115082

**Published:** 2026-08-24

**Authors:** Carlos A Arellanes-Chavez, Alfredo Porras-Mendoza, Jose I Fernández-Faudoa, Ernesto Ramos-Martínez

**Affiliations:** 1 Neurosurgery, Universidad Autónoma de Chihuahua, Chihuahua, MEX; 2 Neurosurgery, Hospital Star Médica, Chihuahua, MEX; 3 Pathology and Laboratory Medicine, Patologia E Inmunohistoquimica de Chihuahua SC, Chihuahua, MEX

**Keywords:** carmustine wafer, ependymal seeding, high-grade glioma (hgg), leptomeningeal dissemination, magnetic resonance spectroscopy (mrs)

## Abstract

Leptomeningeal and ependymal seeding (LES) is an uncommon but devastating complication of high-grade gliomas, and its incidence appears to be rising as improved multimodal treatment prolongs patient survival. Intraoperative ventricular entry and repeated surgery are well-established risk factors, and their coexistence with carmustine wafer implantation raises technical and diagnostic questions that remain underexplored in the literature. We report the case of a 37-year-old man with a right parieto-occipital WHO grade III anaplastic astrocytoma, initially treated with gross total resection and standard chemoradiotherapy. Following local recurrence at 19 months and a second resection with cranioplasty, the patient developed advanced tumor progression 17 months later that required a third surgery, during which an incidental opening of the lateral ventricle occurred; the defect was sealed with Gelfoam (Pfizer Inc., New York, United States) and Surgicel (Ethicon, Inc., Raritan, New Jersey, United States), and eight carmustine wafers were subsequently implanted in the surgical bed. Definitive histopathology documented malignant transformation to glioblastoma (WHO grade IV) with mutant* IDH1*. Two months later, the patient developed acute neurological deterioration with diffuse ependymal and leptomeningeal enhancement; because lumbar puncture was relatively contraindicated, magnetic resonance spectroscopy (MRS) directed at the hypothalamic and cervical regions of abnormal enhancement showed a pattern highly suggestive of active neoplastic seeding (choline/N-acetylaspartate (Cho/NAA) 8.0, choline/creatine (Cho/Cr) 3.2). Despite high-dose corticosteroid therapy, the patient died of progressive motor and respiratory decline. This case illustrates the diagnostic dilemma that arises when intraoperative ventricular entry and subsequent carmustine wafer implantation coincide in the same procedure: distinguishing true leptomeningeal tumor seeding from carmustine-induced chemical leptomeningitis, since both can produce an indistinguishable enhancement pattern on conventional MRI. We also discuss the clinical rationale for using carmustine wafers as a local therapeutic bridge. At the same time, a patient awaits formal oncologic evaluation, a wait that is often prolonged in publicly funded health systems. We review the reported evidence on risk factors for cerebrospinal fluid (CSF) dissemination, carmustine-related complications near the ventricular system, the role of MRS when lumbar puncture is contraindicated, and the relevance of the 2021 WHO nomenclature for interpreting this case.

## Introduction

High-grade gliomas remain among the primary central nervous system neoplasms with the poorest prognosis, with a natural history marked by nearly universal local recurrence. However, as multimodal treatment, including maximal safe resection, radiotherapy, and temozolomide, has prolonged survival, a less common but clinically devastating pattern of progression has emerged with increasing frequency: dissemination of the tumor through the cerebrospinal fluid (CSF), with leptomeningeal and/or ependymal seeding distant from the primary site [1].

Leptomeningeal dissemination of high-grade gliomas was first documented nearly a century ago, and although autopsy series suggest an incidence of up to 20-30% for subclinical spinal seeding, symptomatic disease is far less common, and its clinical recognition is often delayed because its manifestations overlap with other treatment-related complications [2].

Among the surgical factors most consistently linked to leptomeningeal dissemination is intraoperative opening of the ventricular system. Contemporary series based on magnetic resonance imaging (MRI) with prolonged follow-up have identified ventricular entry as the most robust independent risk factor for developing leptomeningeal metastasis in high-grade gliomas, with hazard ratios exceeding eight in some multivariate analyses [2].

In this context, the use of carmustine wafers (Gliadel® Wafer; Azurity Pharmaceuticals, Inc., Woburn, Massachusetts, United States) as local intracavitary chemotherapy adds further complexity. The product's prescribing information explicitly warns that any communication between the resection cavity and the ventricular system must be closed before implantation, given the risk of wafer migration into the ventricular system and obstructive hydrocephalus; inflammatory and infectious complications, including severe cerebral edema and eosinophilic meningitis, have also been described in association with the implant, and these can clinically and radiologically mimic active neoplastic seeding [3-5].

This report describes the case of a young patient with an anaplastic astrocytoma who, after multiple surgeries, experienced incidental ventricular entry followed by carmustine wafer implantation during the same intervention in which malignant transformation to glioblastoma was documented. The patient subsequently developed acute neurological deterioration with imaging findings compatible with diffuse ependymal and leptomeningeal seeding, in a setting where diagnostic lumbar puncture was relatively contraindicated because of the risk of herniation. The aim of this report is twofold: first, to document an uncommon case that simultaneously combines the principal recognized risk factors for CSF dissemination; second, to discuss the particular diagnostic dilemma that arises when factors such as ventricular entry, carmustine implantation, multiple surgeries, and documented malignant transformation coexist, complicating the distinction between true neoplastic seeding and complications related to local treatment.

Beyond its rarity, this case merits report for several reasons. Few published cases document ventricular entry, carmustine wafer implantation, and histologically confirmed malignant transformation converging within a single surgical episode, limiting current understanding of their cumulative contribution to CSF dissemination risk. The coexistence of ventricular entry and carmustine implantation also raises a diagnostic question that, to our knowledge, has not been explicitly addressed in the literature: how to distinguish true leptomeningeal tumor seeding from carmustine-induced chemical leptomeningitis when both can produce an overlapping clinical and radiological picture. This report further illustrates the practical value of directing magnetic resonance spectroscopy (MRS) at multiple, anatomically distant sites of enhancement when lumbar puncture cannot be safely performed, and highlights the clinical relevance of applying current WHO nomenclature to avoid diagnostic imprecision that could affect prognostic counseling. Thus, this case offers educational and clinical value beyond its epidemiological rarity.

## Case presentation

Initial presentation and first diagnosis

A 37-year-old man with no significant past medical history was diagnosed with an anaplastic astrocytoma (WHO grade III) located in the right parieto-occipital region. Gross total surgical resection was performed, followed by standard adjuvant therapy consisting of radiotherapy with concomitant and maintenance temozolomide. The patient maintained an excellent functional status, with a Karnofsky Performance Status (KPS) of 100%, for 19 months.

Recurrence and second surgery

Seven months later, local tumor recurrence was documented. The patient underwent a second surgical resection, with cranioplasty using a methylmethacrylate plate to reconstruct the bone defect. Histopathology reconfirmed the diagnosis of WHO grade III anaplastic astrocytoma.

Advanced tumor progression and third surgery

Seventeen months after this, the patient presented with hemicranial headache, pulsatile tinnitus, and left homonymous hemianopsia. Gadolinium-enhanced MRI showed advanced tumor progression, characterized by an 8-cm occipital cystic lesion with irregular nodular enhancement and a 12 × 11 mm satellite thalamic lesion, with no evidence of ventricular seeding at that time. Displacement of the previously placed methylmethacrylate plate was also noted.

**Figure 1:**
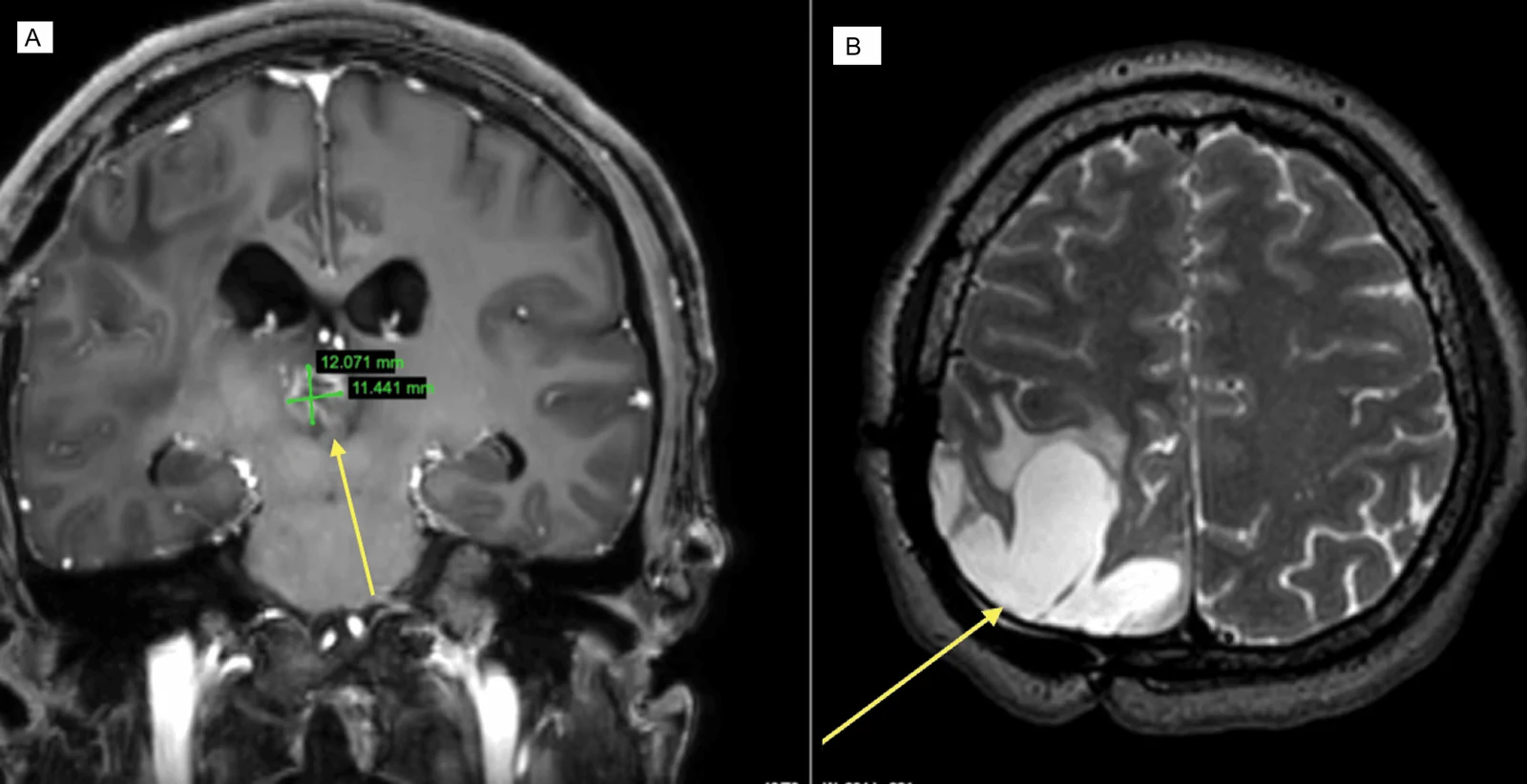
Preoperative images during the third recurrence. (A)* *Contrast-enhanced T1-weighted magnetic resonance imaging (MRI) showing a 12 x 11 mm satellite lesion with ring enhancement, located in the right optic thalamus (yellow arrow). Notably, the ventricular system remains free of tumor involvement or ependymal enhancement; (B) Axial T2-weighted view showing a large, expansive 8 cm cystic lesion in the right occipital region (yellow arrow).

A third surgical intervention was indicated, with the goals of removing the unstable cranioplasty material, evacuating the cystic component, and resecting the satellite thalamic lesion. During dissection, an intraoperative opening of the lateral ventricle occurred, which was sealed with Gelfoam (Pfizer Inc., New York, United States) and Surgicel (Ethicon, Inc., Raritan, New Jersey, United States). Eight carmustine wafers (Gliadel) were subsequently implanted in the surgical bed, and a titanium mesh was placed to close the bone defect.

**Figure 2:**
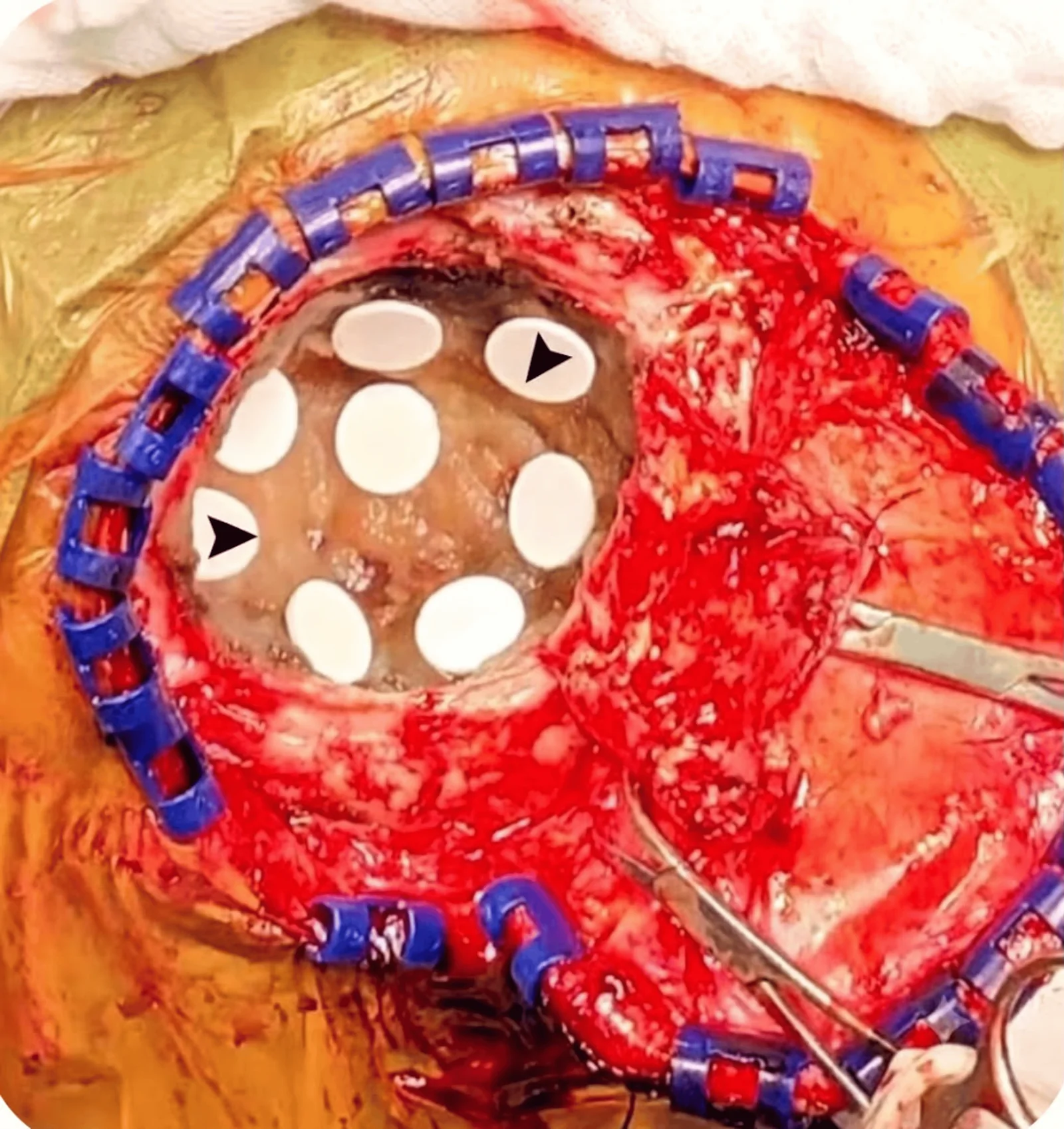
Intraoperative image: placement of carmustine wafers in the surgical bed (black arrowheads).

Histopathologically documented malignant transformation

Definitive histopathological analysis of the surgical specimen confirmed malignant transformation of the tumor to glioblastoma (WHO grade IV). The immunohistochemical profile showed positivity for glial fibrillary acidic protein (GFAP), mutant p53, loss of ATRX expression, *IDH1* mutation, and a Ki-67/MIB-1 proliferation index of 60%.

Acute neurological deterioration and findings of dissemination

Two months after the third surgery, the patient was admitted with acute neurological deterioration, presenting with somnolence, positive meningeal signs, left hemiparesis (3/5), and a KPS of 40%. Whole-neuraxis MRI revealed diffuse ependymal enhancement throughout the ventricular system, basal and hypothalamic leptomeningeal enhancement, and cervical arachnoiditis with nodular intradural enhancement, findings compatible with extensive tumor seeding via the CSF.

**Figure 3:**
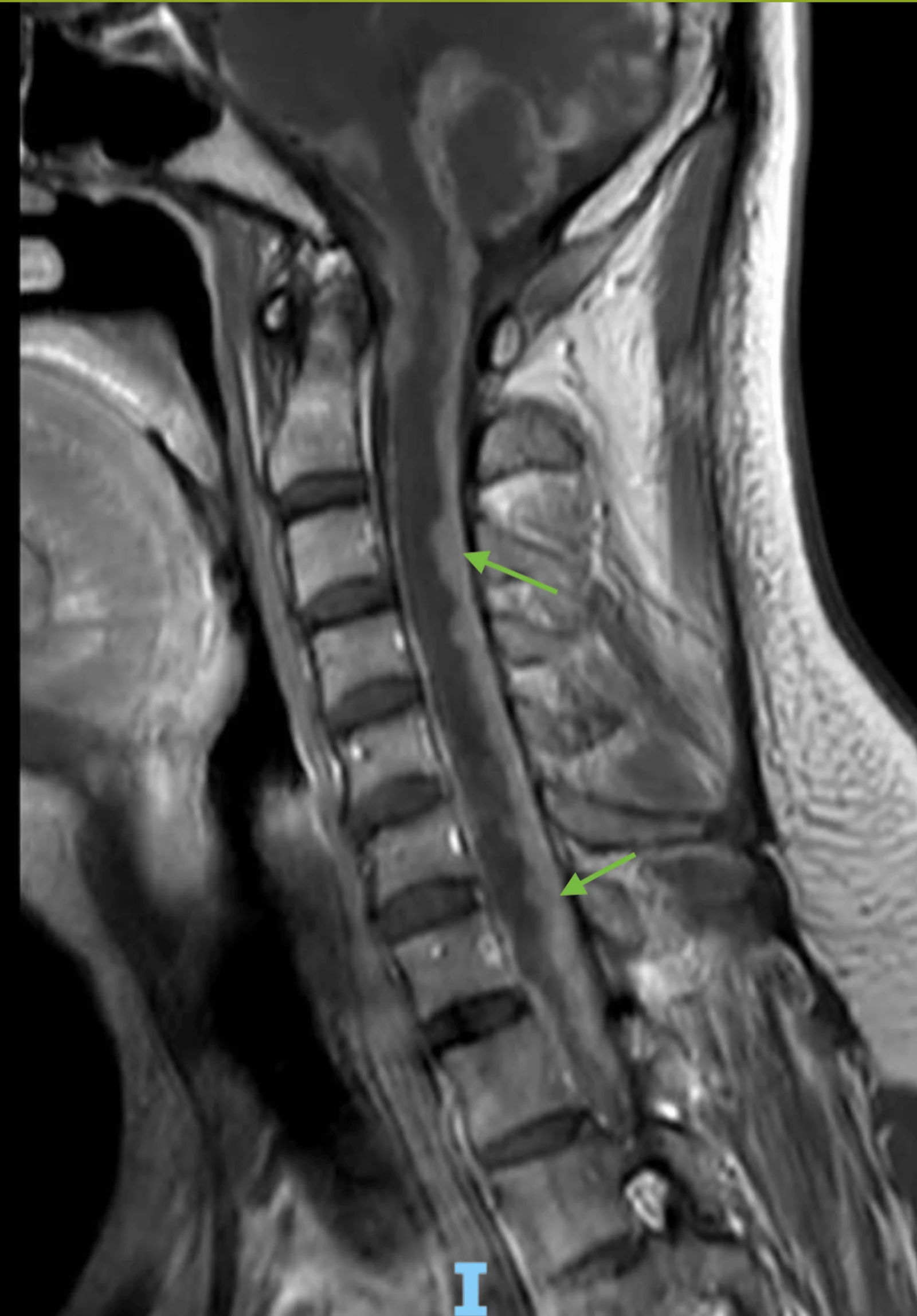
Cranial-cervical MRI, sagittal view with contrast medium. Diffuse cervical spinal arachnoiditis with nodular intradural enhancement along the spinal cord (green arrows).

These findings immediately raised the central diagnostic question in this case: distinguishing true leptomeningeal tumor dissemination from chemical leptomeningitis secondary to the carmustine wafer implantation performed two months earlier, given that both can produce a diffuse leptomeningeal and ependymal enhancement pattern that is virtually indistinguishable on conventional MRI.

Given the risk of herniation associated with the clinical and radiological picture, diagnostic lumbar puncture was deferred, and MRS directed at the areas of abnormal enhancement was performed instead. The study showed a metabolic pattern highly suggestive of active neoplastic seeding, with a choline/N-acetylaspartate (Cho/NAA) ratio of 8.0 and a choline/creatine (Cho/Cr) ratio of 3.2. Spectroscopy was directed specifically at the regions with abnormal enhancement most representative of the observed dissemination pattern, including the hypothalamic region and the cervical region with arachnoiditis and nodular intradural enhancement, in order to characterize the metabolic profile at sites of seeding distant from one another and from the original surgical bed.

**Figure 4:**
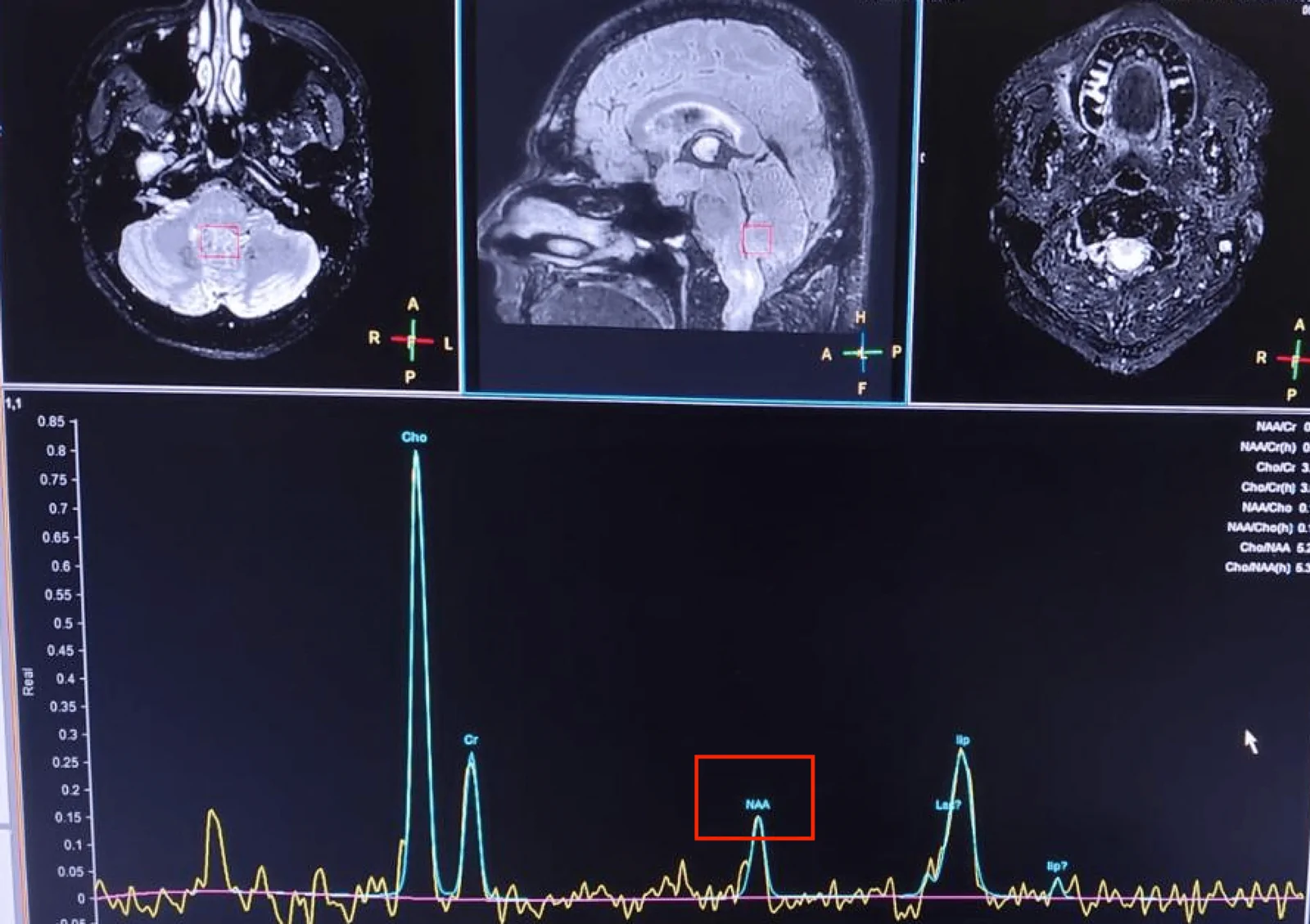
Advanced neuroimaging via magnetic resonance spectroscopy (MRS) Multivoxel MRS targeted at the area of basal leptomeningeal enhancement (small red squares). The metabolic profile shows an absolute choline (Cho) concentration of 0.8, N-acetylaspartate (NAA) of 0.1 (big red square), and creatine (Cr) of 0.25, yielding a markedly elevated Cho/NAA ratio of 8.0 and a Cho/Cr ratio of 3.2. While traditionally indicative of high-grade neoplastic turnover, this aggressive metabolic blueprint represents tissue remodeling and necrosis induced by local carmustine toxicity.

Clinical course and outcome

Empiric management with high-dose dexamethasone was initiated, resulting in transient improvement in hemiparesis. However, over the following days, the patient developed progressive motor deterioration culminating in quadriplegia, followed by respiratory compromise that resulted in death.

## Discussion

Epidemiology and biology of leptomeningeal and ependymal dissemination in high-grade gliomas

CSF dissemination of high-grade gliomas is a clinically underrecognized entity. In the largest available series with prolonged imaging follow-up, leptomeningeal metastasis was documented in 11% of patients with high-grade glioma, approximately two-thirds of whom were symptomatic [2]. Overall survival after diagnosis of dissemination was markedly lower than in patients without this complication (median 239 vs 626 days), underscoring its prognostic relevance. The fourth ventricle has been identified as the preferred site for seeding, owing to the multidirectional CSF flow dynamics within that cavity. However, in the present case, the pattern was diffuse, involving the ventricular system in its entirety, the basal and hypothalamic leptomeninges, and the cervical intradural space.

Intraoperative ventricular entry as a risk factor for CSF dissemination

According to the available evidence, surgical opening of the ventricular system is the single most consistent risk factor for the development of leptomeningeal dissemination in high-grade gliomas. In a cohort study of 239 patients, ventricular entry was associated with an 8.1-fold increase in the risk of leptomeningeal metastasis and was documented in 100% of patients who subsequently developed this complication after open surgery [2]. A subsequent study of 200 patients with glioblastoma found that the risk of postoperative leptomeningeal dissemination in the presence of ventricular entry was 27.4%, significantly higher than in cases without ventricular entry (4%) [6]. Other described risk factors include ependymal invasion, tumor fragmentation in contact with CSF, and, notably for this case, multiple reinterventions, which are associated with higher risk owing to repeated tumor manipulation and selection of more aggressive tumor phenotypes after successive courses of chemoradiotherapy. The patient described here presented several of these factors simultaneously: three successive surgeries, documented ventricular entry during the last intervention, and concurrent malignant transformation, configuring a cumulative risk profile that is infrequently described together in the literature.

Clinical rationale for carmustine wafer implantation as a therapeutic bridge during postoperative recovery

Beyond the technical considerations related to closure of the ventricular communication, the decision to implant carmustine wafers at the third surgery also responded to a practical clinical need: providing immediate local chemotherapy in the surgical bed during the period in which the patient heals the surgical wound and recovers from the procedure, an interval during which systemic chemotherapy and further radiotherapy typically cannot be started. This strategy becomes particularly relevant when considering the time that may elapse before formal evaluation by the oncology service, which is sometimes considerably delayed because of high demand for care, a situation that is compounded in publicly funded health systems, where saturated oncology clinics and limited appointment availability can prolong the wait between surgery and the first oncologic assessment by several weeks. In this context, carmustine wafer implantation not only serves a local therapeutic purpose in its own right, but also acts as a pharmacological bridge that maintains some degree of disease control while the patient completes surgical wound healing, a period during which other treatment modalities are contraindicated or not practicable, and awaits the oncologic evaluation that will define the subsequent adjuvant treatment plan. This practical consideration must nevertheless be weighed against the technical and inflammatory risks discussed below, particularly in a scenario such as this patient's, with recent ventricular communication.

Carmustine wafer implantation in the presence of ventricular communication: technical considerations and an added dilemma

The prescribing information for carmustine wafers explicitly states that any communication between the resection cavity and the ventricular system larger than the diameter of a wafer should be closed before implantation, in order to reduce the risk of wafer migration into the ventricular system and secondary obstructive hydrocephalus [5]. In the case presented, the ventricular opening was sealed intraoperatively with Gelfoam and Surgicel before the eight wafers were implanted, in keeping with this recommendation. However, beyond the mechanical risk of migration, the literature has documented inflammatory complications associated with carmustine implantation that can clinically and radiologically mimic tumor progression or neoplastic dissemination, further complicating the interpretation of subsequent findings in a patient with concomitant ventricular entry.

Among these complications, severe cerebral edema, occasionally with fatal outcome, has been attributed to a local inflammatory reaction to the implant [3], as well as eosinophilic meningitis triggered by the wafers, with subacute onset of headache, vomiting, and altered consciousness weeks after surgery [4]. These entities share with true leptomeningeal dissemination a clinical picture of subacute neurological deterioration with inflammatory findings on neuroimaging, which demands a high index of suspicion and, when possible, the search for additional data, such as that provided by MRS, that may help guide the differential diagnosis without necessarily resorting to high-risk invasive procedures in the acute setting.

The diagnostic dilemma: limitations of lumbar puncture and the utility of MRS

The central diagnostic question running through this report is whether the acute neurological deterioration with diffuse leptomeningeal and ependymal enhancement corresponded to true leptomeningeal tumor dissemination or to a chemical leptomeningitis induced by the carmustine wafers implanted two months earlier. Both entities share a similar time course after surgery, a nearly superimposable enhancement pattern on conventional neuroimaging, and a clinical presentation dominated by subacute neurological deterioration, so their differentiation requires additional diagnostic tools when, as in this case, CSF cytology cannot be safely obtained.

The historical diagnostic standard for leptomeningeal dissemination is CSF cytology obtained by lumbar puncture; however, its sensitivity is limited, ranging from 50% to 60% even in the presence of overt symptomatic disease, with false-negative rates that improve only partially by increasing sample volume, processing it immediately, and repeating sampling [7]. Furthermore, studies comparing gadolinium-enhanced MRI with CSF cytology have found imperfect concordance between the two tests, so that neither alone can be considered fully conclusive [8]. In the present case, lumbar puncture was relatively contraindicated because of the risk of herniation given the acute neurological deterioration and the findings of diffuse enhancement with possible mass effect, which led the treating team to defer this procedure and instead use MRS as a complementary noninvasive diagnostic tool.

MRS provides metabolic information that can help differentiate active neoplastic tissue from other causes of leptomeningeal or ependymal enhancement. Characteristically, high-grade tumor tissue shows elevated choline, a marker of cell membrane proliferation, with a marked reduction or absence of NAA, reflecting loss of viable neuronal tissue; elevated Cho/Cr ratios have been consistently associated with higher tumor grade and even with the ability to differentiate high-grade glioma from metastasis [9,10]. In the current patient, the values obtained (Cho/NAA of 8.0 and Cho/Cr of 3.2) are well above the ranges usually reported even for non-disseminated high-grade gliomas, in which average Cho/Cr ratios typically fall between 2 and 2.6 [9,10], which was highly suggestive of diffuse neoplastic activity rather than a purely inflammatory or postsurgical process. Although MRS does not replace cytological or histopathological confirmation, in scenarios where lumbar puncture carries an unacceptable risk, it constitutes a reasonable tool for guiding urgent clinical decision-making.

In this case, the ability to direct spectroscopy to both the hypothalamic region and the cervical region proved particularly valuable, as it allowed documentation of a metabolic profile compatible with active neoplastic activity at two anatomically distant sites, both remote from the original surgical bed, which reinforced the interpretation of a diffuse CSF dissemination process rather than an inflammatory phenomenon confined to the periwafer area. The concordance of elevated Cho/NAA and Cho/Cr ratios at both hypothalamic and cervical locations provided an additional argument against chemical leptomeningitis strictly localized to the implant site, although it did not entirely rule it out as a concurrent component.

Relevance of the 2021 WHO nomenclature for the interpretation of the case

An additional point that merits discussion is the histopathological terminology used. Since publication of the fifth edition of the World Health Organization Classification of Tumors of the Central Nervous System (WHO CNS5) in 2021, the term “glioblastoma” has been reserved exclusively for *IDH*-wildtype tumors; tumors with grade 4 histology but with an IDH mutation, such as the one described in this patient, with *IDH1* mutation documented by immunohistochemistry, are now formally classified as *IDH*-mutant astrocytoma, WHO grade 4, rather than glioblastoma [11,12]. This distinction is not merely semantic: grade 4 *IDH*-mutant astrocytomas represent a biologically and prognostically distinct entity from *IDH*-wildtype glioblastoma, with a typically more prolonged natural history and a tendency to arise, as in this case, from progressive malignant transformation of a lower-grade tumor in a young patient. The histopathology report for this case used the term “grade IV glioblastoma” in keeping with pre-2021 nomenclature, still widely used in clinical practice; however, for the sake of diagnostic precision, communication with multidisciplinary teams, and eventual inclusion in registries or clinical trials, it is worth noting that, according to current criteria, the correct diagnosis would be *IDH*-mutant astrocytoma, WHO grade 4. This clarification illustrates a second layer of the “diagnostic dilemma” in this case: not only the difficulty of distinguishing neoplastic seeding from treatment-related complications, but also the need for precise histomolecular nomenclature to appropriately guide prognosis and treatment decisions.

Leptomeningeal dissemination in high-grade gliomas in the literature

Leptomeningeal dissemination of high-grade gliomas has been the subject of a growing number of case reports and retrospective series over the past two decades, reflecting both greater clinical recognition and the prolonged survival now afforded by contemporary multimodal treatment. The most extensive review available on the topic, which analyzed 155 articles published between 1989 and 2019, describes a heterogeneous entity in terms of clinical presentation and time course, with a median survival after diagnosis of leptomeningeal dissemination of only a few months despite the various therapeutic strategies attempted [13]. In an institutional series of glioblastoma patients treated with contemporary regimens, leptomeningeal dissemination was identified in a minority of cases, and patients who developed it showed significantly shorter overall survival than those without this complication, with no treatment modality, such as intrathecal chemotherapy, craniospinal radiotherapy, or symptomatic management, evaluated, substantially altering the disease course [14]. A Korean analysis of risk factors and outcomes in patients with confirmed leptomeningeal dissemination reported similar findings, with post-diagnosis survival typically under six months [15].

Individual case reports illustrate the diversity of presentations described. Cases of extensive leptomeningeal and spinal dissemination during early adjuvant chemoradiotherapy have been documented, with rapidly progressive motor deterioration similar to that observed in the patient described here [16], cases of acute tetraplegia as the initial manifestation of leptomeningeal dissemination in a multicentric *IDH*-wildtype glioblastoma [17], transient responses with temozolomide and bevacizumab regimens in recurrent disseminated disease, without sustained impact on survival [18], and even atypical presentations of glioblastoma manifesting primarily as diffuse leptomeningeal dissemination without an identifiable dominant parenchymal lesion, underscoring the breadth of the clinical spectrum of this complication [19]. Taken together, the available literature agrees that, regardless of the triggering mechanism, ventricular entry, multiple reinterventions, malignant transformation, or, as discussed in this report, coexistence with carmustine wafer implantation, established leptomeningeal dissemination in high-grade gliomas carries a grim prognosis that has changed little despite advances in the systemic treatment of localized disease.

Prognosis and therapeutic limitations of disseminated disease

The prognosis of established leptomeningeal and ependymal dissemination in high-grade gliomas is grim, with reported median survivals of only a few months after diagnosis even with active treatment. Transient clinical and radiological responses have been described with temozolomide and bevacizumab in isolated cases of recurrent glioblastoma dissemination, although without sustained impact on survival [18], and other reports of extensive dissemination with neuraxis involvement have documented a rapidly progressive clinical course similar to that of the patient described here, with ascending motor deterioration and a fatal outcome within weeks to a few months [16,17]. In the case presented, the combination of an extensive disseminated tumor burden at diagnosis, an already deteriorated functional status (KPS 40%), and the absence of viable additional surgical or radiotherapeutic options limited therapeutic possibilities to symptomatic corticosteroid management, which did not alter the disease course beyond a transient improvement.

## Conclusions

This case illustrates the uncommon convergence of the principal recognized risk factors for CSF dissemination in high-grade gliomas, multiple surgeries, intraoperative ventricular entry, and malignant transformation, together with carmustine wafer implantation in a surgical bed with recent ventricular communication. This combination produced a diagnostic picture in which true leptomeningeal tumor seeding could not be confidently distinguished from carmustine-induced chemical leptomeningitis on clinical and radiological grounds alone. MRS directed at two anatomically distant sites of enhancement provided concordant metabolic evidence of diffuse neoplastic activity, supporting seeding over a purely local inflammatory process. Finally, the documented *IDH1* mutation formally reclassifies this tumor as an *IDH*-mutant grade 4 astrocytoma rather than a glioblastoma under current WHO nomenclature, a distinction with direct prognostic and management implications.
